# Genome-Wide Identification and Analysis of the *WRKY* Transcription Factor Family Associated with Leaf Senescence in Alfalfa

**DOI:** 10.3390/plants13192725

**Published:** 2024-09-29

**Authors:** Xiaojing Peng, Jinning Hu, Xiangxue Duan, Maofeng Chai, Jiangqi Wen, Zengyu Wang, Hongli Xie

**Affiliations:** 1 Shandong Key Laboratory for Germplasm Innovation of Saline-Alkaline Tolerant Grasses and Trees, Key Laboratory of National Forestry and Grassland Administration on Grassland Resources and Ecology in the Yellow River Delta, College of Grassland Science, Qingdao Agricultural University, Qingdao 266109, China; 2 Qingdao Key Laboratory of Specialty Plant Germplasm Innovation and Utilization in Saline Soils of Coastal Beach, College of Grassland Science, Qingdao Agricultural University, Qingdao 266109, China; 3 College of Animal Science and Technology, Qingdao Agricultural University, Qingdao 266109, China; 4 Institute for Agricultural Biosciences, Oklahoma State University, Ardmore, OK 73401, USA

**Keywords:** *WRKY*, leaf senescence, *Medicago sativa*, expression profile

## Abstract

Leaves are the most significant parts of forage crops such as alfalfa. Senescence is the terminal stage of leaf development and is controlled by an integrated myriad of endogenous signals and environmental stimuli. WRKY transcription factors (TFs) play essential roles in regulating leaf senescence; however, only a few studies on the analysis and identification of the *WRKY* TF family in *Medicago Sativa* have been reported. In this study, we identified 198 *WRKY* family members from the alfalfa (*M. sativa* L.) cultivar ’XinjiangDaye’ using phylogenetic analysis and categorized them into three subfamilies, Groups I, II, and III, based on their structural characteristics. Group II members were further divided into five subclasses. In addition, several hormone- and stress-related cis-acting elements were identified in the promoter regions of *MsWRKYs*. Furthermore, 14 aging-related *MsWRKYs* genes from a previous transcriptome in our laboratory were selected for RT-qPCR validation of their expression patterns, and subsequently cloned for overexpression examination. Finally, *MsWRKY5*, *MsWRKY66*, *MsWRKY92*, and *MsWRKY141* were confirmed to cause leaf yellowing in *Nicotiana benthaminana* using a transient expression system. Our findings lay a groundwork for further studies on the mechanism of *M. sativa* leaf aging and for the creation of new germplasm resources.

## 1. Introduction

WRKY transcription factors (TFs) are a type of sequence-specific DNA-binding proteins predominantly identified in plants [1,2]. The WRKY TF family members are characterized by a highly conserved WRKYGQK amino acid sequence and a C2H2 or C2HC zinc finger-like motif [2]. WRKY TFs are categorized into three groups depending on the number of WRKY sequences (two in group I, one in groups II and III) and the structure of the zinc finger-like motif (group III WRKY TFs have modified C2HC motifs) [1]. Group II WRKY proteins can be further classified into five subgroups (II-a, II-b, II-c, II-d, and II-e) based on their primary amino acid sequences [2]. In 1994, the first member of the WRKY gene family, *SPF I*, was identified in *Ipomoea batatas* and was shown to be triggered by sucrose and polygalacturonic acid [3]. Subsequently, an increasing number of *WRKY* gene families have been identified and studied in the genomes of model plants, including *Arabidopsis thaliana* [1], *Medicago truncatula* [4], maize [5], wheat [6], and *Trifolium pratense* L. [7].

Plants may encounter a variety of biotic and abiotic stresses during their growth and development. With the increase in climate change and extreme weather events, the proportion of abiotic stresses affecting crop production is growing. According to statistics, global yield loss of major crops due to various abiotic stresses is estimated to be around 50% [8]. Many transcription factors (TFs) contribute to plant resistance and tolerance to unfavorable environmental conditions, making them potential candidate genes for breeding. Among these TFs, WRKY transcription factors stand out. They regulate the expression of abiotic stress-responsive genes or directly control the expression of these genes by activating or repressing the transcription of downstream genes. WRKYs play key roles in abiotic and biotic growth and development [9]. Leaf senescence is the final stage of leaf development, which is a highly orchestrated process regulated by endogenous and environmental signals [10]. Leaf senescence is characterized by three main features: degradation of chlorophyll and chloroplasts, disruption of protein homeostasis, and induction of genes related to senescence [11]. Senescence-related traits are closely associated with yield and stress tolerance [12]. Expression profiling indicates that numerous *WRKY* genes are highly activated during senescence and they make up the second-largest category of transcription factor genes in the senescence transcriptome, indicating the key role of WRKY TFs in leaf senescence [13].

To date, several *WRKY* genes have been identified as key regulatory factors in Arabidopsis leaf senescence: Arabidopsis WRKY47 actively controls age-triggered leaf senescence by activating the programmed cell death-related genes *BFN1* and *MC6* [14], WRKY1 positively regulates leaf senescence by inducing SA biosynthesis genes [15], and WRKY45 interacts with the DELLA protein RGL1 [16]. Additionally, WRKY6 [17], WRKY22 [18], WRKY26 [19], WRKY42 [20], WRKY53 [21], WRKY55 [22], WRKY71 [23], and WRKY75 [24] positively regulate leaf senescence, whereas WRKY54 and WRKY70 [25] negatively regulate leaf senescence by delaying it. WRKY activation in crops against abiotic stresses also helps improve plant resilience and ensures crop yields even in challenging conditions [26,27]. WRKY TFs have also been documented to be involved in leaf senescence in several different plant species. In tobacco, NtWRKY70b triggers the expression of the ROS-synthesis-related gene *NtRbohD* and the chlorophyll degradation gene *NtPPH*, contributing to the positive regulation of leaf senescence. Additionally, in wheat, if the transcription of *TaWRKY13-A* is impaired, leaf senescence is delayed [28]. In sorghum, SbWRKY50 directly binds to the promoters of several chlorophyll catabolic genes and negatively regulates chlorophyll degradation, thus delaying leaf senescence [29].

Premature leaf senescence caused by abiotic stresses can significantly impact the yield and quality of alfalfa. However, the WRKY family is not well studied in *Medicago Sativa*. Alfalfa (*M. sativa* L.), known as the “queen of forages”, is considered one of the most important forage crops in the world [30] and is widely used as a high-quality legume forage due to its high yield, good palatability, and high nutritional value [31]. Leaves, as a primary harvesting organ, are a limiting factor affecting the yield and quality of *M. sativa*. Premature induction of leaf senescence can severely limit plant biomass [32]. Therefore, preventing or delaying premature senescence to increase biomass accumulation could improve the quality and economic benefits of alfalfa [33].

In this study, we first characterized the WRKY family members in the *M. sativa* genome through bioinformatics analysis. We then identified candidate *WRKY* genes involved in leaf senescence by analyzing the transcriptomes of *M. sativa* leaves subjected to darkness and salt stress. Similar approaches have been used in *M. truncatula* [34] to screen senescence-related genes and lay the foundation for subsequent studies on the regulatory mechanisms of *M. sativa* leaf senescence, as well as for breeding and selecting high-quality *M. sativa* germplasm resources.

## 2. Results

### 2.1. Gene Identification and Phylogenetic Tree Construction

**Figure 1:**
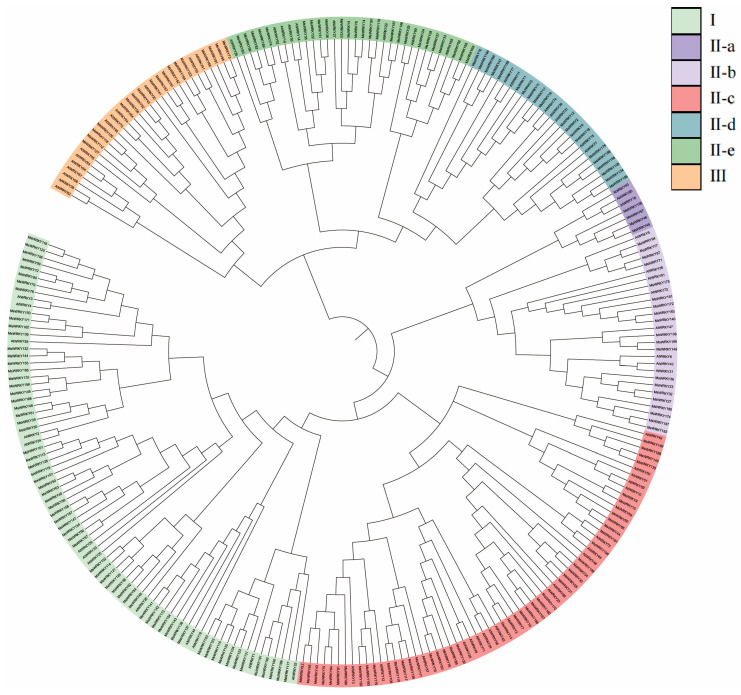
A neighbor-joining phylogenetic tree was constructed using MEGA11.0 software with 1000 boot-strap replications, by comparing WRKY TFs from *M. sativa* L. (Ms) and *Arabidopsis thaliana* (At). Various highlighted colors correspond to the different subgroups.

The protein sequences of the WRKY TFs family from the Arabidopsis database were used to query the Pfam database to obtain the conserved domain (PF03106) file for WRKY proteins. The ‘hmmsearch’ command was executed with parameters set to E valuae ≤ 1 × 10^-10^ and bits ≥ 85. A total of 198 MsWRKYs were identified in the *M. sativa* genome. Based on their chromosomal positions, they were named MsWRKY1 through MsWRKY198 (Table S1). Next, MEGA11.0 was used to conduct clustering analysis of the 72 Arabidopsis proteins and 198 putative MsWRKY proteins, using 1000 bootstrap replicates for statistical reliability (Figure 1). The 198 WRKY TFs were categorized into three groups, Group I, Group II, and Group III, (Figure 1), with 66, 114, and 18 WRKY members, respectively. Group II was further divided into five subclasses: Group II-a, -b, -c, -d, and -e. Group II-a, with only 4 MsWRKYs, had the least members among all subclasses. Groups II-b and II-d had 20 MsWRKYs each, Group II-c had 45, and Group II-e had 25. Since the functions of numerous Arabidopsis WRKYs have been investigated, it is possible to make inferences about the functions of MsWRKYs that are grouped with Arabidopsis WRKYs by considering previous research.

### 2.2. Sequence and Structural Analysis of MsWRKYs

**Figure 2:**
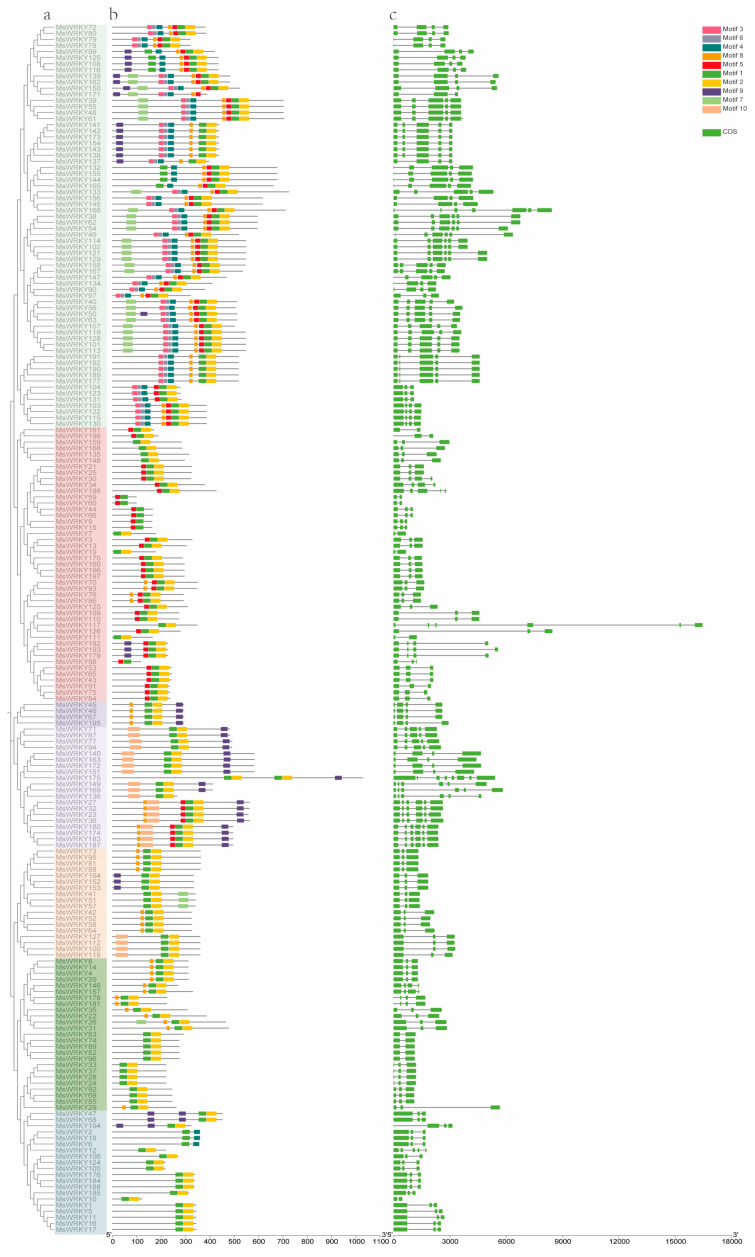
Analysis of phylogenetic relationships, motifs, and gene structure of WRKY TFs from *M. sativa*. (**a**) Phylogenetic tree of 198 MsWRKYs in *M. sativa*. The colors highlighted the different subgroups are same as that in Figure 1. (**b**) Conserved motif arrangements of MsWRKYs. The motifs are highlighted in various colored boxes. Motif 1 represents the WRKY domain. (**c**) Exon-intron organizations of *MsWRKYs*. Green boxes indicate exons; black lines indicate introns.

A phylogenetic tree was created using MEGA11.0 to examine the structure and evolutionary connections of the *MsWRKY* genes. The conserved motifs were identified by utilizing the Multiple Em for MEME online tool, and the MsWRKY protein sequence as a reference “https://meme-suite.org/meme/ (accessed on 26 August 2024)” with the parameters set to zero or one motif per sequence, a maximum of 10 motifs, and an optimum motif width ranging from 4 to 14. Motifs with an e-value < 1 × 10^−10^ were retained for further analysis. Based on the genome Gff3 annotation file, the structure of the *MsWRKY* genes was determined. Gene Structure View (Advanced) of TBtools (v2.038) software was utilized to generate phylogenetic trees, motifs, and gene structure diagrams (Figure 2). It can be seen that *MsWRKYs* in group I have 7–9 types of motifs, *MsWRKYs* in group II have 2–6 types of motifs, and *MsWRKYs* in group III have 3 types of motifs. Correspondingly, *MsWRKYs* in group I contain 3–7 exons, *MsWRKYs* in group II contain 2–8 exons, and *MsWRKYs* in group III contain 3 exons. In brief, within the same group or subgroup, the distribution of conserved motifs as well as exon-intron organization were similar, indicating that genes within the same group or subgroup may have similar biological functions.

### 2.3. Cis-Acting Elements of MsWRKYs

**Figure 3:**
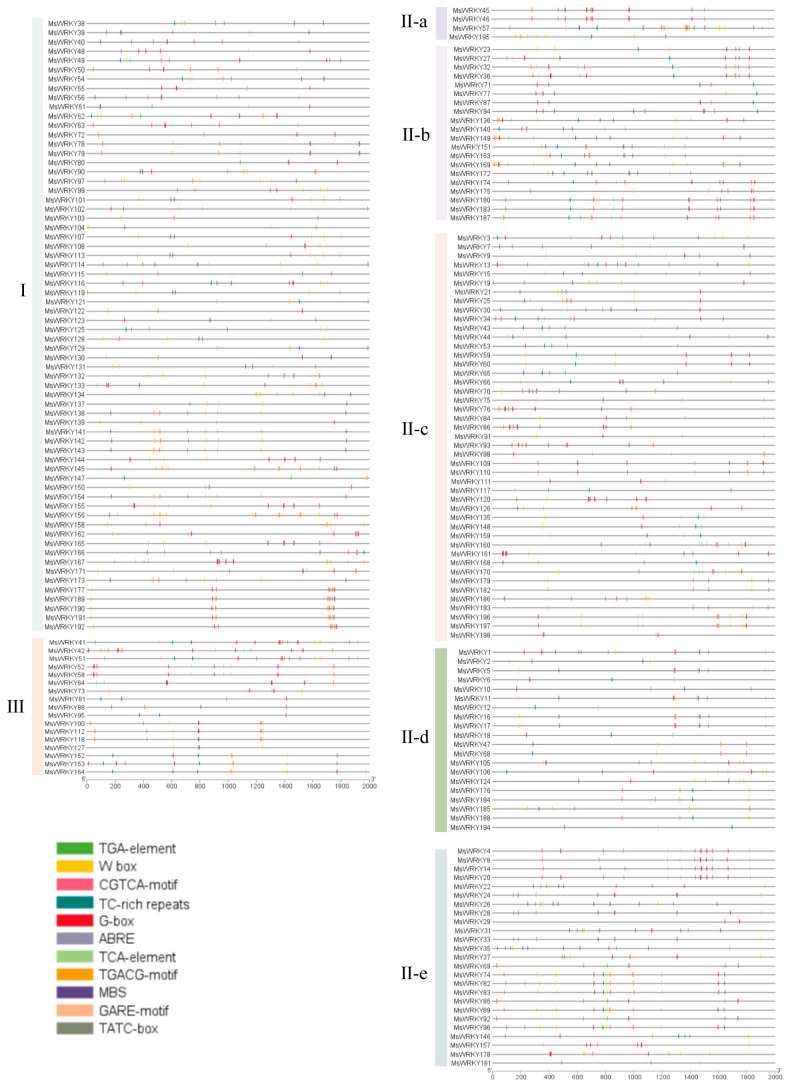
The 2 kb promoter sequences of the *MsWRKY* gene contain various *cis*-acting elements. Different colored rectangles indicate different *cis*-elements, positioned according to their locations within the promoters.

Using the GXF Sequences Program in TBtools, the promoter sequences were obtained by determining the 2000 bp upstream sequences of the start codons of the *MsWRKY* genes. Using the PlantCARE database to predict potential *cis*-acting elements, we identified 11 types of *cis*-acting elements in 198 gene promoter regions, including two stress response elements (MBS, and TC-rich repeats), seven hormone response elements (ABRE, CGTCA motif, GARE motif, TATC box, TCA element, TGACG motif, and TGA element), the G-box element known as transcription factors (NAC, BHLH and bZIP) binding motif, and the W-box element of the WRKY transcription factor-binding site (Figure 3). 164 MsWRKYs contain light-responsive cis-acting elements (G-box), 152 MsWRKYs contain abscisic acid-responsive elements (ABRE), 143 MsWRKYs contain methyl jasmonate acid-responsive elements (CGTCA motif and TGACG motif), 120 MsWRKYs contain salicylic acid-responsive cis-acting elements (TCA-elements), 85 MsWRKYs contain MYB Binding Site (MBS) and 81 have TC-rich repeats. Each of the 198 MsWRKYs has at least two elements in the promoter region, with *MsWRKY57*containing 48 elements, the highest number among all *MsWRKY* genes. The majority of *MsWRKY* genes contain one or more W-box elements in the promoter region.

### 2.4. Verification of Screened Leaf Senescence-Related WRKY Genes

**Figure 4:**
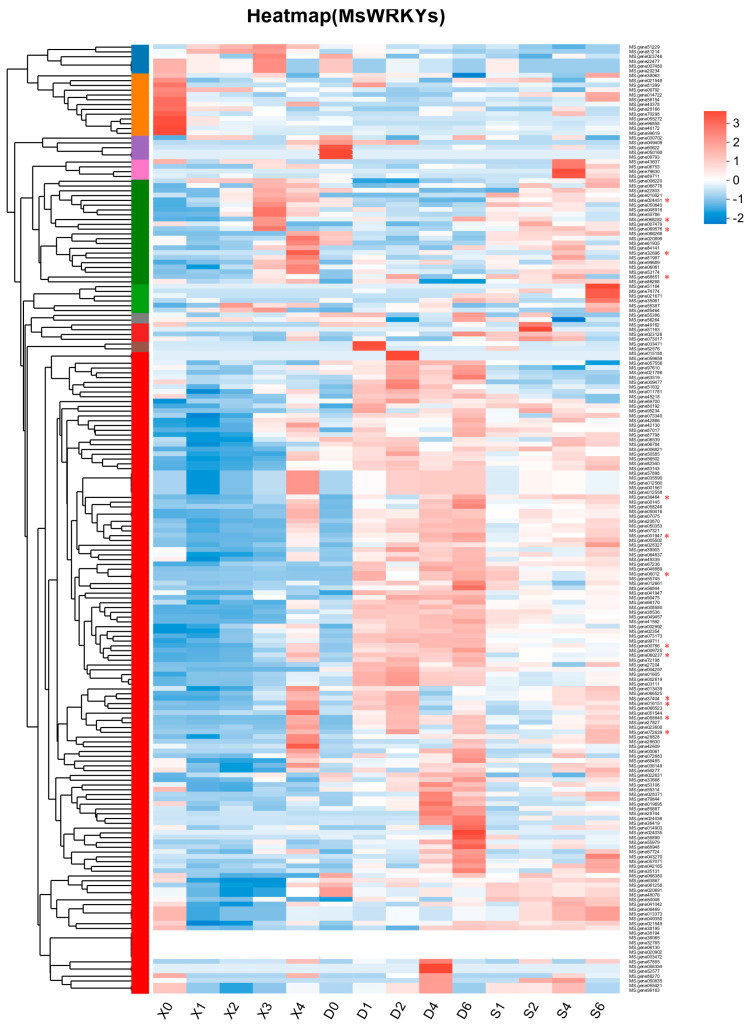
A heatmap displaying the RNA−Seq data for 198 *MsWRKYs*, with expression levels normalized by row using the Z−Scores algorithm. The color scale on the right of the heatmap shows relative expression, with the color gradient from blue to red indicating increased expression levels. X0 (top not fully unfolded leaf), X1 (top fully unfolded first leaf), X2 (top fully unfolded second leaf), X3 (top fully unfolded third leaf), X4 (bottom leaf with senescent symptom); D0, D1, D2, D4, D6 (leaves treated in the dark for 0, 1, 2, 4 and 6 days); S1, S2, S4, S6 (leaves treated with salt for 1, 2, 4 and 6 days). Fourteen genes selected for RT-qPCR were labeled with an asterisk.

Expression data of 198 *MsWRKYs* were downloaded from the laboratory’s previously determined leaf senescent transcriptome (Table S2) and used to create a heat map (Figure 4). We examined the genes that were highly expressed in X3, X4 relative to X0, and the genes highly expressed in D4, D6, S4, and S6 relative to D0. We selected top five genes from each of these six comparisons based on the fold upregulation of their expression levels for subsequent experiments. A BLAST search on the alfalfa website was performed and the top-ranked genes without 100% homology were retained, resulting in a final selection of 14 *MsWRKY* genes for further analysis (Figure 4; Table S3).

**Figure 5:**
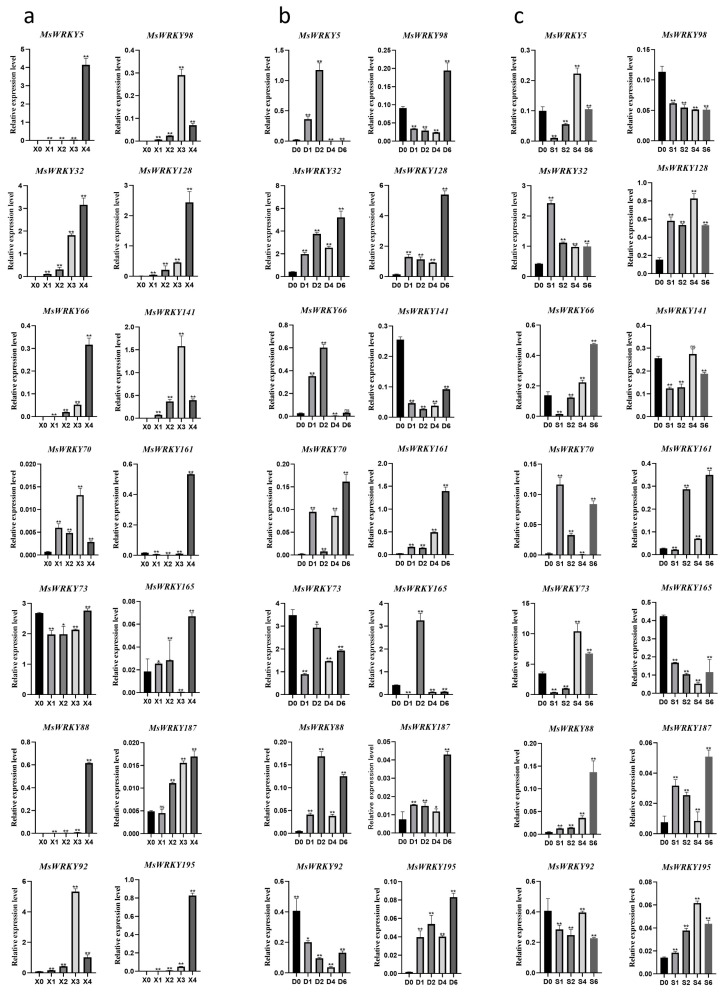
RT-qPCR results of 14 *MsWRKYs* in the process of leaf senescence under (**a**) natural condition (X0, X1, X2, X3, X4 represent different stages of leaf development), (**b**) dark stress (D0, D1, D2, D4, D6 represent 0, 1, 2, 4, and 6 days of dark treatment), (**c**) salt stress (S1, S2, S4, S6 represent 1, 2, 4, and 6 days of the 150 mM NaCl treatment). The error bars indicate the standard deviation of three biological replicates. Relative expression was calculated using the 2^–ΔCT^ method. The data for gene expression are presented as the mean ± SD and were analyzed to detect significant differences by ANOVA using GraphPad Prism 8 (NS: not significant; * *p* < 0.05; ** *p* < 0.01) against D0 or X0.

The expression levels of these 14 genes at different leaf developmental stages and under different treatment conditions were verified by RT-qPCR, and the results were presented as column charts (Figure 5). With the exception of *MsWRKY165*, the other 13 genes showed trends consistent with that in the RNA-seq (Figure 4 and Figure 5). The qRT-PCR results revealed that *MsWRKY5*, chosen from the X4vsX0 comparison, was also induced by dark treatment and salt treatment at D1, D2 and S4 (Figure 5a–c). *MsWRKY66*, selected from the X3vsX0 comparison, was also up-regulated when exposed to salt treatment and showed significant up-regulation at the S6 period (Figure 5c). Similarly, *MsWRKY92* and 141 exhibited significant changes in expression when subjected to dark and salt treatments.

### 2.5. Verification of Gene Function Using Transient Expression

**Figure 6:**
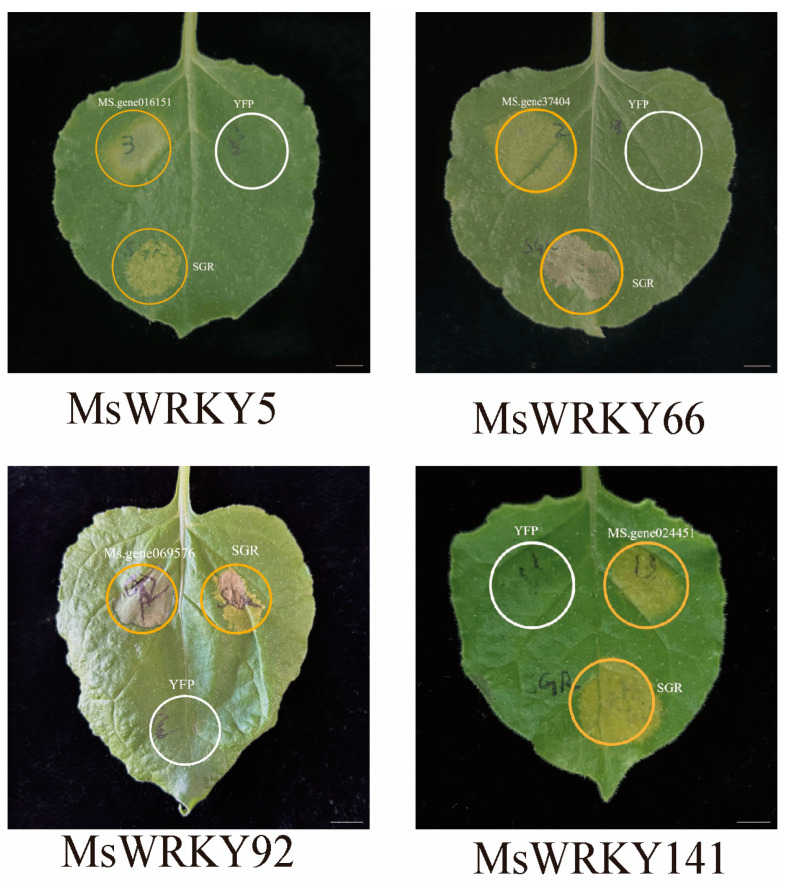
Functional validation of selected *MsWRKYs* was conducted using an *Agrobacterium*-mediated transient expression assay. Symptoms of leaf senescence in representative *Nicotiana benthamiana* leaves appeared after infiltration with various constructs encoding *MsWRKY5*, *MsWRKY66*, *MsWRKY92*, *MsWRKY141*, *MsSGR* and an empty vector with YFP. Positive control: SGR. Negative control: empty vector with YFP. Bar = 1 cm.

*Agrobacterium*-mediated transient gene expression was performed in *Nicotiana benthaminana* leaves to quickly screen for *M. sativa* senescence-associated TFs (SA-TFs). The coding regions of the selected SA-TFs were amplified from cDNA derived from senescing *M. sativa* leaves using gene-specific primers, the PCR products were cloned into the pFGC-eYFP vector at the Bam HI site, and the sequence-verified constructs were then transformed competent *Agrobacterium* EHA105 cells. When the *Agrobacterium* cultures reached OD600 = 0.6 to 0.8, 3–4 week-old *N. benthaminana* plants were infiltrated. As *MsSGR* promotes leaf senescence [35], *MsSGR* was used as a positive control, and an empty vector was used as a negative control. After infiltration, the leaves were kept in the dark for one day, followed by normal light conditions. Phenotype observations were conducted 2–3 days later. The transient expression of *MsWRKY5*, *MsWRKY66*, *MsWRKY92*, and *MsWRKY141* in *N. benthaminana* resulted in a significant premature aging phenotype, similar to the phenotype induced by infiltration of the *MsSGR* gene in alfalfa (Figure 6). These results suggest that these four *MsWRKY* genes play important roles in regulating leaf senescence in *M. sativa*.

## 3. Discussion

The WRKY transcription factor family is an important regulator of leaf senescence and one of the most extensively studied transcription factors [36]. Although it has been observed and researched in various species, it has not been documented in *M. sativa*. In this study, we conducted a bioinformatics analysis of 198 WRKY gene family members found in the alfalfa cultivar “Xinjiang Daye”. To determine the evolutionary relationships between the *MsWRKY* gene family, we constructed a phylogenetic tree of the *WRKY* gene family and investigated the gene structure and motif distribution. We also predicted the *cis*-acting elements of the *WRKY* gene family.

We identified 198 WRKY genes from the *M. sativa* genome, and quantitatively the number of WRKY genes in *M. sativa* was similar to that of tetraploid soybean [37], as well as significantly higher than that of diploid *M. truncatula* [4]. A total of 93 WRKY transcription factors were identified in *M. truncatula*, and the number identified in *M. sativa* was nearly twice that of *M. truncatula*. These indicate that the *MsWRKY* gene family is evolutionarily conserved in *M. sativa* in the context of gene number. The construction of a phylogenetic tree of the *MsWRKY* gene family revealed their evolutionary relationship; subsequently, the genetic structure and base distribution of the 198 *MsWRKYs* were analyzed. The phylogenetic analysis showed that the 198 MsWRKYs could be divided into three subgroups according to their evolutionary relationships. MsWRKY5, MsWRKY66, and MsWRKY92 are members of subclasses 2-d, 2-c, and 2-e, respectively, whereas MsWRKY141 is a member of Group 1. AtWRKY06 [17], AtWRKY42 [20], AtWRKY45 [16], AtWRKY47 [14], AtWRKY71 [23], AtWRKY75 [24], and AtWRKY22 [18] in Group 2, and AtWRKY01 [15] and AtWRKY26 [19] in Group 1 play a role in regulating leaf senescence. It can be inferred that MsWRKYs in the same subgroup with AtWRKYs are likely involved in the regulation of leaf senescence, laying the foundation for further research on the functions of MsWRKYs and the mechanisms of leaf senescence regulation. Conserved motif analysis revealed that all of these MsWRKYs contained motif 1, which was identified as WRKYGQK. This explains why members of the same subgroup share similar gene structures and motifs, supporting the phylogenetic results.

*Cis*-acting elements are DNA sequences present upstream of a gene, and they are involved in regulating its expression. They do not encode proteins but provide binding sites for transcription factors and other regulatory factors [38]. In this study, we used PlantCARE to predict *cis*-acting elements 2000 bp upstream of the start codon of the 198 *MsWRKY* genes. The promoter regions of the 198 *MsWRKY* genes were investigated for 11 types of elements, including two stress-responsive elements, seven hormone-responsive elements, one light-responsive elements G-box and one WRKY transcription factor-binding site W-box element. As the core sequence of the W-box is “TGAC”, the W-box can be used for predicting the target genes of WRKY TFs [39]. We found that 143 out of 198 *MsWRKY* genes contain two or more W-box elements in the promoter region, while 164 out of 198 *MsWRKY* genes have the important light-responsive cis-acting element known as G-box in their promoter. In plants, many genes related to photosynthesis and photomorphogenesis contain G-box element. G-box is able to respond to the environmental changes in light, temperature, and moisture leading to changes in the expression of related genes. This enables plants to adapt to different environmental conditions. Furthermore, at least 2 *cis*-acting elements were found in the promoter region of each *MsWRKY* gene. The cis-acting elements in the promoter region of the gene suggest that the gene may perform different biological functions under varying stress conditions. Studies on *cis*-regulatory elements are important for further understanding plant defense responses to abiotic and biotic stresses [40].

WRKYs play a central role in growth and development [9], including leaf senescence, the final stage of leaf growth and development [41]. They are the main players in mediating defense responses in plants [42]. In this study, we verified the expression patterns of 14 *MsWRKYs* by RT-qPCR and subsequently determined their function by overexpressing them in tobacco. The results showed that four *MsWRKYs*, namely *MsWRKY5*, *MsWRKY66*, *MsWRKY92*, and *MsWRKY141*, exhibited the same leaf yellowing phenotype as the positive control *MsSGR*. We hypothesized that these four MsWRKYs may be involved in the regulation of leaf senescence in *M. sativa*.

Leaf senescence is the final stage of leaf development and is crucial for the fitness of the plant [43]. Inhibiting the expression of *MsSGR* in *M. sativa* can increase the crude protein content in hay by 2.3–5.5% and retain more than 50% of chlorophyll, greatly improving the quality of alfalfa hay [35]. In nature, plants often experience various stresses, such as salinity stress and insufficient light. These stresses can lead to premature leaf senescence. This is why we are studying WRKY genes, which are regulated by darkness and salt stress and are related to leaf senescence. In the future, silencing or mutating these WRKY genes may also improve forage quality, similar to silencing SGR.

The results of this study establish a foundation for future research into the roles and regulatory mechanisms of the *WRKY* gene family in *M. sativa* leaf senescence. These studies suggest that targeting specific WRKY transcription factors may offer a novel approach to manipulate leaf senescence in *M. sativa*, potentially enhancing yield and stress tolerance. In the future, more deep understanding of the interplay between WRKY transcription factors and other hormonal or signaling pathways may lead to biotechnological strategies that fine-tune senescence for better crop management.

## 4. Materials and Methods

### 4.1. Plant Material and Treatment

The plant materials of *M. sativa* ‘Xinjiang Daye’ and *Nicotiana benthamiana* were grown as described previously [44]. Briefly, *M. sativa* seeds were placed in petri dishes and covered with wet filter paper, once the radicle grew to 3–4 cm, the seedlings were then transferred to Hoagland’s nutrient solution for hydroponic cultivation; *Nicotiana benthamiana* seeds germinated in pots. All plants were grown in an artificial climate chamber (16 h light/8 h dark, temperature 22 °C; 65% relative humidity; 150 µmol·m^−2^·s^−1^ light intensity).

When *M. sativa* ‘Xinjiang Daye’ plants were 4 weeks old, the first leaf that has not fully unfolded was collected and regarded as the first stage of leaf development (X0). Subsequent leaves were collected and identified as X1, X2, and X3 according to leaf position, with X4 representing the leaves with senescent symptom in the lower part of the plant. The compound leaves of the third leaf position from apex were collected and treated with darkness or salt for 0, 1, 2, 4, and 6 days in a culture dish. The darkness treatment solution was half-strength Murashige–Skoog containing 3 mM MES buffer, pH of 5.8; while the salt treatment solution is further supplemented with 150 mM NaCl.

Leaves with three biological replicates at different stages of development (X0, X1, X2, X3, X4) and at different treatment times (0, 2, 4, and 6d) were collected for RNAseq and RT-qPCR analysis.

### 4.2. Differential Expression Analysis

A differential expression analysis of genes was performed to identify differentially expressed genes between samples and to study their functions. To identify genes associated with leaf senescence, the expression levels of each transcript were determined using the transcripts per million reads method. The significantly differentially expressed genes were identified using DESeq2 [45] according to the following criteria: FDR < 0.05 and |log2FC| ≥ 1.

### 4.3. Sequence and Phylogenetic Analysis

In this study, we looked up the protein sequences of the WRKY gene family according to the Arabidopsis thaliana database and searched the Pfam database “https://pfam.xfam.org/ (accessed on 26 August 2024)” using their protein sequences as templates, downloaded the file of conserved structural domains of WRKY proteins (PF03106), and executed the hmmsearch command, in which E value ≤ 1 × 10^−10^, while bits ≥ 85, and finally a total of 198 MsWRKYs were identified in *M. sativa*. TBtools (v2.038) software was used to visualize the gene structure and motif distribution [46]. Conserved motifs were analysed for MsWRKYs using the MEME online website, with parameters set to zero or one motif per sequence, up to a maximum of 10 motifs. A total of 72 Arabidopsis WRKY sequences from The Arabidopsis Information Resource (TAIR) and 198 MsWRKY sequences from *M. sativa* were used to construct a phylogenetic tree. MEGA 11.0 was used to build phylogenetic trees using the neighbor-joining method, through applying the Poisson model, pairwise deletion, and 1000 bootstrap replications [47]. PlantCARE was used to predict the *cis*-acting elements of 2000 bp sequences upstream of the start codon of 198 MsWRKYs [48], and TBtools was used to visualize *cis*-acting elements.

### 4.4. Quantitative Polymerase Chain Reaction (RT-qPCR)

To identify the expression patterns of *MsWRKYs*, RNA was extracted from samples at each time point and analyzed using RNA-seq. RT-qPCR was performed for verification [49]. Total RNA was extracted from leaf samples at each stage using the Takara MiniBEST Plant RNA Extraction Kit (Plant RNA Purification Reagent for plant tissue; Invitrogen Corp., Carlsbad, CA, USA.) and reverse transcribed into cDNA using HiScript III^®^ RT SuperMix for qRT-PCR (+gDNA wiper) from Vazyme Biotech Co., Ltd. in Nanjing, China. RT-qPCR was conducted using the ChamQ SYBR Color qRT-PCR Master Mix (Vazyme Biotech Co., Ltd., Nanjing, China). *MsUBC* was used as the reference gene. Relative expression was calculated using the 2^–ΔCT^ method. The gene expression data is presented as the mean ± SD and was analyzed to detect significant differences using ANOVA in GraphPad Prism 8 (NS: not significant; * *p* < 0.05; ** *p* < 0.01). Each experiment was performed in three biological replicates, with each biological replicate being run in triplicate. Primers were designed using the NCBI database, and the primer sequences are listed in Table S3.

### 4.5. Verification of Gene Function through Transient Expression

Selected genes were cloned for transient expression in *Nicotiana benthamiana*, with *MsSGR* as a positive control. Using cDNA as a template, selected target genes were cloned and sequenced to verify the sequence accuracy. Target genes were ligated into the pFGC-eYFP vector using seamless DNA cloning (ClonExpress R Ultra One Step Cloning Kit; Vazyme Biotech Co., Ltd.). The recombinant plasmid was transformed into *Escherichia coli* DH5α (Vazyme Biotech Co., Ltd.) for amplification and detection, and positive colonies were selected and verified by sequencing. The plasmid was then extracted and transformed into *Agrobacterium tumefaciens* strain GV3101. A single positive *A. tumefaciens* colony was selected for infiltration into *N. benthamiana* using a previously described method [50]. Specific amplification primers were designed using CE Design software “http://www.vazyme.com (accessed on 26 August 2024)”, and the sequences are listed in Table S4.

## 5. Conclusions

In this study, we characterized MsWRKY transcription factors through genome-wide identification of the WRKY gene family in *M. sativa*, utilizing sequence homology and conserved WRKY domains to classify them into various groups based on their structure and phylogenetic relationships. An expression analysis revealed differential expression of several WRKY genes during *M. sativa* leaf senescence. Functional studies in *N. benthaminana* suggested the involvement of specific WRKY proteins in regulating senescence-associated phenotypes. Moreover, some senescence-associated *MsWRKY* genes responded to various abiotic stresses, such as salinity, indicating a dual role in stress resilience and senescence regulation. Collectively, these findings emphasize the complex roles of WRKY transcription factors in *M. sativa* leaf senescence and their potential as targets for enhancing crop resilience and productivity. Further investigations in this area will continue to elucidate the molecular mechanisms underlying senescence, offering a new path for the genetic enhancement of alfalfa and other forage crops.

## Data Availability

The original contributions presented in the study are included in the article/Supplementary Materials, further inquiries can be directed to the corresponding author.

## Supplementary Materials

The following supporting information can be downloaded at: https://www.mdpi.com/article/10.3390/plants13192725/s1. Table S1. Basic information of *MsWRKYs* gene. Table S2. Expression levels for the 198 *MsWRKYs* screened genes. Table S3. Sequences of primers used in qRT-PCR. Table S4. Sequences of primers used in PCR.
